# Hypolipidemic Activity of Olive Oil-Based Nanostructured Lipid Carrier Containing Atorvastatin

**DOI:** 10.3390/nano12132160

**Published:** 2022-06-23

**Authors:** Heba S. Elsewedy, Tamer M. Shehata, Mervt M. Almostafa, Wafaa E. Soliman

**Affiliations:** 1 Al Bilad Bank Scholarly Chair for Food and Security in Saudi Arabia, The Deanship of Scientific Research, The Vice Presidency for Graduate Studies and Scientific Research, King Faisal University, Alhofuf 36362, Saudi Arabia; tshehata@kfu.edu.sa (T.M.S.); malmostafa@kfu.edu.sa (M.M.A.); weahmed@kfu.edu.sa (W.E.S.); 2 Department of Pharmaceutical Sciences, College of Clinical Pharmacy, King Faisal University, Alhofuf 36362, Saudi Arabia; 3 Department of Chemistry, College of Science, King Faisal University, Alhofuf 36362, Saudi Arabia; 4 Department of Biomedical Sciences, College of Clinical Pharmacy, King Faisal University, Alhofuf 36362, Saudi Arabia; 5 Department of Microbiology and Immunology, Faculty of Pharmacy, Delta University for Science and Technology, Gamasa, Mansoura 11152, Egypt

**Keywords:** hypolipidemic, obesity, olive oil, atorvastatin, nanostructured lipid carrier

## Abstract

Currently, hyperlipidemia is a growing health issue that is considered a risk factor for obesity. Controlling body weight and modifying life style in most of cases are not adequate and the condition requires medical treatment. Statin drugs (mainly Atorvastatin (ATO)), have been used broadly and for long time as medications for handling higher levels of lipid, especially bad cholesterol, which accordingly controls the prevalence of obesity. Still, the obstacle that stands in front of any formulation is the poor solubility of the drug. Low solubility of ATO came up with poor absorption as well as poor bioavailability. This paved the way for the present study, which aimed to exploit nanotechnology and develop certain nanolipid carriers that could accommodate hydrophobic drugs, such as ATO. Nanostructured lipid carrier (NLC) containing ATO was fabricated using olive oil. Olive oil is natural plant oil possessing confirmed hypolipidemic activity that would help in improving the efficacy of the formulation. Via applying the Quality by Design (QbD) approach, one NLC formula was selected to be optimized based on appropriate size and higher entrapment. Optimized ATO-NLC was scrutinized for zeta potential, in vitro study and kinetic profile. Moreover, stability testing and in vivo hypolipidemic behavior was conducted. The optimized NLC formulation seemed to show particle size (254.23 nm) with neutral zeta potential (−1.77 mV) and entrapment efficiency (69.56%). The formulation could be prolonged for 12 h and provided higher % of release (97.17%). Stability testing confirmed the role of modifying the surface of the formulation with PEG-DSPE in providing a highly stable formulation that could withstand three months storage in two altered conditions. Ultimately, optimized ATO-NLC could successfully lower total cholesterol level in rats induced with obesity and fed a high-fat diet. Remarkably, ATO-NLC prepared with olive oil, in addition to shielding its surface, would provide a stable formulation that holds up the synergistic action between olive oil and ATO.

## 1. Introduction

Obesity is an excessive accumulation of body fat, which not only affects fitness and well-being but also influences public health issues leading to various health problems [1]. In recent decades, the prevalence of obesity has increased all over the world and given rise to a wide variety of complications, which could be one of the leading causes of death. One could suffer from obesity due to several reasons, including lack of exercise, improper diet, environmental factors or genetic factors [2]. Presently, obesity increases the probability of several complications, such as diabetes, high blood pressure, stroke and coronary heart diseases [3]. Consequently, the treatment of obesity provides effective help in managing weight as well as monitoring the most related consequences of obesity, such as hyperglycemia and hyperlipidemia.

Hyperlipidemia is a disease burden impressively associated with obesity and characterized by the presence of extraordinary fats in the blood serum. The lipid profile in our blood is generally expressed by the presence of fats in the blood, such as cholesterol and triglycerides. Normal cholesterol levels most likely range between 200 and 240 mg/dL, since below 200 mg/dL is considered as desirable blood cholesterol; however, above 240 mg/dL is categorized as high blood cholesterol [4]. Cholesterol is commonly classified into two kinds, high density lipoprotein (HDL) and low-density lipoprotein (LDL), which are renowned as good and bad cholesterol, respectively. Hypercholesterolemia, which is a sign of an elevated blood cholesterol level, is a very critical issue that has to be handled wisely otherwise it could cause severe heart diseases [5]. Additionally, a correlation was established between hypercholesterolemia and certain pathological conditions, which exhibited a likely prognosis of the existing outbreak of COVID-19 [6]. Therefore, maintaining appropriate levels of cholesterol is essential for avoiding the risk of such diseases; otherwise, a protocol of treatment has to be followed.

One of the extensively spread protocols for managing most impaired health issues is the manipulation of natural products [7]. The benefit of using these natural compounds is their natural origin that ensures efficacy and the least side effects [8]. Natural products have been used for treating a variety of diseases, such as cancer, bacterial infection, inflammatory disorders, controlling blood lipid profile and others [9,10]. Plant oils are natural products extracted from plants and attract significant attention due to their effectiveness in treating several diseases [11]. Olive oil is a natural oil, obtained from the Olea europea L fruit, belonging to the family Oleaceae [12]. It consists mostly of triacylglycerols and free fatty acids in addition to minor components, such as phytosterols and tocopherols [13]. The main component of triacylglycerols is signified by monounsaturated fatty acid, such as oleic acid, which is responsible for the nutritional value and health functions of olive oil [14]. Olive oil is reported to have a significantly valuable influence as an antioxidant, anti-inflammatory, anti-bacterial, anti-platelet and anticancer agent, in addition to hypoglycaemic and hypocholesterolemic properties [12]. The mechanism by which olive oil could lower the cholesterol level in blood is attributed to its content, mainly monounsaturated fat, which reduces the chance of blocking the arteries with lipids [15]. Moreover, another study suggested that the hypolipidemic effect of olive oil returns the abundant anti-oxidant component, polyphenols, which play a crucial role in decreasing cholesterol oxidation, to consequently inhibit its accumulation on the walls of the blood vessels [16]. Interestingly, olive oil has been documented to have lipid lowering capacity enough to be used alone [17] or in combination with other hypolipidemic drugs, such as Atorvastatin (ATO). Such investigations emphasized that combining olive oil with a low dose of ATO would provide the same plasma lipid profile similar to that obtained from higher doses of ATO but with minor adverse reactions [18].

ATO is a synthetic statin and one of the recommended therapies for hyperlipidemia [19]. In addition to its ability to manage cardiovascular disorders, it is frequently used for lowering LDL and triglycerides and raising HDL levels [20]. Its action depends on inhibiting a certain enzyme known as the HMG-CoA reductase enzyme that are present in the liver and has a major role in cholesterol production [21]. According to the Biopharmaceutical Classification System (BCS), ATO belongs to class II (BCS-II), which means it has poor solubility and high permeability [22]. ATO water solubility was detected to be 0.1 mg/mL, [23] while it was 8.25 mg/mL in olive oil [24,25]. It showed a pKa value of 4.5 [26] and log *p* value of 4.06, indicating its lipophilicity [27]. Since it exhibited poor water solubility, it shows poor oral bioavailability, which is only 12% [22]. Therefore, improving ATO solubility and bioavailability is essential and could be achieved via entrapment of the drug into different nanocarriers that would provide a prospective drug delivery as well.

Nano-systems are branches of the nanotechnology platform that has recently prompted great concern due to the ability of enhancing solubility, bioavailability, offering long term stability and controlled drug release properties [28]. A lipid-based nanocarrier is a nano-system that has the ability to encapsulate hydrophobic compounds owing to its characteristic of colloidal dispersion. Several systems are regarded as lipid-based nanocarriers, such as liposomes, nanoemulsions, solid lipid nanoparticles (SLN) and nanostructured lipid carriers (NLC). NLC is the adapted form of SLN; however, its lipid phase consists of both liquid lipid (oil) and solid lipid (fat) [29]. The ability of NLC to encapsulate hydrophobic compounds into the solid lipid phase gives rise to higher loading capacity, better entrapment of the drug and better physical stability if compared to SLN [30].

The previous literature investigated the incorporation of ATO in NLC, as in Ghanem et al. However, the main objective was to compare different experimental designs for optimizing the prepared Atorvastatin nanostructured lipid carriers [31]. Moreover, Elmowafy et al. demonstrated the dominance of NLCs for improving the oral bioavailability of ATO [32]. In light of the previous facts, the objective of the current study was raised. Several NLCs as a nanolipid formulation was developed using olive oil and entrapping ATO for enhancing the solubility and magnifying the hypolipidemic activity. As far as we know, this is first NLC formulation incorporating ATO and prepared with olive oil. The Quality by Design (QbD) strategy was implemented via operating the Box Benkhn Design (BBD) approach in order to achieve an optimized formulation with the best attributes. The BBD is a tool for selecting the best optimized formula based on certain investigated factors. The optimized NLC preparation was investigated for its physicochemical properties. Finally, some parameters were measured; namely, total cholesterol (T-Chol), triglycerides (TG), LDL and HDL levels to check and verify the synergistic action between olive oil and ATO.

## 2. Materials and Methods

### 2.1. Materials

ATO calcium was acquired from the Saudi Pharmaceutical Industries and Medical Appliances Corporation (SPIMACO, Al Qassim, Saudi Arabia) as a gift. Olive oil was obtained from Aljouf Agricultural Development Co. (Aljouf, Saudi Arabia). Lauroyl polyoxyl-32-glycerides (Gelucire 44/14^®^) and MC60 glycerol monocaprylocaprate (Labrafac™) were procured from Gattefosse SAS (Saint-Priest Cedex, France). Tween 80 was purchased from Sigma-Aldrich Co. (St Louis, MO, USA). Distearoyl phosphatidylethanolamine-N-[methoxy poly (ethylene glycol)-2000] (PEG-DSPE) was bought from Lipoid LLC (Newark, NJ, USA). Total glycerides, total cholesterol, LDL and HDL cholesterol kits were bought from the United Diagnostic Industry (Dammam, Saudi Arabia). Other chemicals used in the study were of analytical grade.

### 2.2. Preliminary Studies

Fundamentally, the lipid phase of the NLC, formed of Gelucire 44/14^®^, represented the solid lipid phase and olive oil signified the liquid lipid phase. However, a precise ratio should be evaluated in order to ensure the homogenous and miscible phase. Therefore, different ratios of solid lipid and liquid lipid were tried, such as 1:9, 2:8 and 3:7, respectively. Gelucire 44/14^®^ and olive oil were mixed together with the proposed ratios and stirred for 1 h at 70 °C using a shaker water bath (Gesellschaft fur, Labortechnik mbH, D-30938, Burgwedel, Germany). Next, the mixture was left to cool and examined visually for its miscibility or any detected phase separation [33,34].

### 2.3. Experimental BBD

Subsequent to the preliminary study, BBD was operated, which facilitated the assumption of the data via selecting certain independent variables to study their influence on some dependent responses (33). Factorial design was created, in which three factors were investigated at three levels (−1, 0, 1) representing the value of the lowest, central and the highest level of variations as displayed in Table 1. The selected independent variables were concentrations of lipid phase (A), Tween 80 as surfactant (B) and Labrafac™ as co-surfactant (C) along with their effect on two responses, namely particle size Y_1_ and entrapment efficiency (EE) Y_2_. The study was assembled utilizing Design-Expert version 12.0 software (Stat-Ease, Minneapolis, MN, USA), demonstrating some data analysis using analysis of variance (ANOVA) tests. Furthermore, modeling graphs were depicted by the software and confirmed by certain mathematical equations for supplemental explanation [35].

### 2.4. ATO-NLCs Preparation

**Figure 1:**
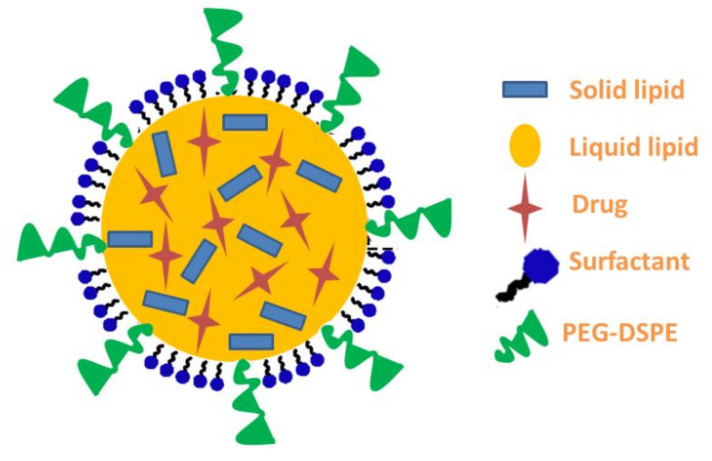
Representing the structure of the prepared ATO-NLC shielded with PEG-DSPE.

As shown in Table 2, BBD supports the development of various NLC formulations with different independent variable concentrations using the melt emulsification–ultrasonication method [36]. An illustrative structure for NLC is shown in Figure 1 representing the mechanism of the development and surface modification with PEG-DSPE. According to the results obtained from the preliminary study, the most proper ratio between solid lipid and liquid lipid phase was applied. Simply, the calculated amount of Gelucire 44/14^®^ was melted at a temperature higher than its melting point (55 °C). Then, olive oil was added to the melted Gelucire 44/14^®^, to form the lipid phase to which, ATO (10 mg), PEG-DSPE (50 mg) and the specific amount of the co-surfactant (Labrafac™) were added. Continuous stirring was applied until a homogenous mixture was achieved. In order to form the emulsion, up to 10 mL of the aqueous phase emulsified with a determined amount of surfactant (Tween 80) was heated at the same temperature and then added slowly to the lipid phase while stirring. The obtained emulsion was subjected to homogenization for 5 min at 10,000 rpm using the Ultra-Turrax homogenizer (IKA-T25; Staufen, Germany). Subsequently, the mixture was sonicated using the probe sonicator XL-200, Qsnonica (Newtown, CT, USA) for 30 s to get a proper particle size [35]. The developed dispersion was kept at room temperature to allow cooling and formation of NLC.

### 2.5. Particle Size Analysis

Almost 5 µL of ATO-NLCs were mixed with 3 mL of distilled water in a cuvette to be analyzed for the particle sizes and corresponding PDI. This study was performed utilizing a Malvern Zetasizer (Nanoseries, zs; Malvern Instruments, Malvern, UK) using the dynamic light scattering technique that was adjusted at 25 °C [37].

### 2.6. EE%

The percentage of ATO trapped inside the NLC formula was evaluated using the centrifugation method utilizing centrifuge (Andreas Hettich GmbH, Co.KG, Westphalia, Germany). In short, a sample of ATO-NLC was loaded into a centrifugation tube such as Amicon^®^ ultra-4 (Ultracel- 10K), then, centrifuged at 4 ºC for 1 h, with a rotation of 4000 rpm. The gathered filtrate, carrying the free drug, was diluted and analyzed spectrophotometrically through a spectrophotometer (U.V. Spectrophotometer, JENWAY 6305, Bibby Scientific Ltd., Staffs, UK) at λ_max_ 246 nm [38]. The percentage of entrapment was calculated as follows:

% EE = ((Total drug − Free drug)/Total drug) × 100

### 2.7. Zeta Potential Measurement

The Zeta potential of particles is a very valuable assessment for emphasizing the physical stability of the formulation. Zeta potential could explain the intensity of the electrostatic repulsions that might exist between particles and avoid their aggregation [39]. Accordingly, the optimized formulation was evaluated for its surface charge using the Malvern Zetasizer (Nanoseries, zs; Malvern Instruments, Malvern, UK) depending on the electrophoretic mobility [40].

### 2.8. Fourier-Transform Infrared Spectroscopy (FTIR) Study

Any probable compatibility between the drug and other ingredients in the formulation could be distinguished via an FTIR spectrophotometer (FTIR spectrophotometer, SHIMADZU, IRAFFINITY-1S, Tokyo, Japan). Briefly, a thin KBr disk was prepared by pressing over to get the plate on which a small amount of the investigated sample was added and left to dry in a vacuum. Different samples were studied at a scale between 4000 and 400 cm^−1^ that was helpful for recording the compound spectra [41]. The studied samples were pure ATO, pure NLC without the drug and optimized ATO-NLC.

### 2.9. In Vitro Study

The percentage of drug released from the prepared NLC and ATO suspension was determined using the Agilent Fiber optics dissolution system (Agilent Technologies, Santa Clara, California, USA). The dissolution media was phosphate buffer pH 6.8, into which a hollow glass tube was immersed. The tube was closed from one side with a cellophane membrane (MWCO 2000–15,000) that was stabilized with bands. An ATO sample of 1 mL was added to the glass tube and allowed to run at 50 rpm while keeping the temperature at 37 ± 0.5 °C. The absorbance of each sample was measured automatically by means of fiber optics attached to the dissolution apparatus that adjusted to analyze at λ_max_ 246 nm at predetermined time intervals (0.25, 0.5, 1, 2 up to 12 h) [42]. The study was performed three times.

### 2.10. Kinetic Study

The kinetic of Ator release from the fabricated NLC formulation was studied by applying various kinetic models where the relation between the drug concentration versus time was plotted. The profile of the release was fitted to zero-order, first order, Higuchi Kinetics and Korsmeyer–Peppas model. Regarding the zero-order model, it showed a linear relation between concentration and time. However, the first order equation exhibited a linear correlation between log concentrations against the time. The kinetic release would obey the Higuchi equation if a linear relation was plotted between drug concentration versus the square root of time (t^0.5^). On the other hand, the Korsmeyer–Peppas equation illustrates the linear graph between log concentration against log-time [43].

### 2.11. Stability Study

The stability study is a very important test for the developed formulation to determine its strength and efficacy. Therefore, the optimized ATO-NLC formulation was kept in a closed container and examined for its stability following 1 and 3 months at different storage conditions, room temperature (25 ± 2 °C) and at refrigeration (5 ± 2 °C). The study was carried out in accordance with the recommendations of the International Conference on Harmonization (ICH). The evaluated parameters include particle size and in vitro release [11].

### 2.12. In Vivo Study

#### 2.12.1. Animals

Our study required twenty-five male Wister rats of average weight (200 ± 25 g). All the rats were acquired from the Experimental Animal Research Centre at King Saud University, Riyadh, Saudi Arabia. Rats were housed under suitable laboratory conditions at 25 ± 2 °C with appropriate humidity (70%) while keeping a light/dark cycle every 12 h.

#### 2.12.2. Ethical Statement

The handling of animals and the protocol of the experimental study were executed in agreement with the guidelines of ethical conduct for animal use at King Faisal University, Al-ahsa, Saudi Arabia. Additionally, permission was given by the Research Ethics Committee (REC) of King Faisal University, approval number (KFU-REC-2022-MAR-EA000530).

#### 2.12.3. Induction of Obesity

For obesity induction, animals have to be fed a certain diet to provide them with high calories, such as the high-fat rodent diet (HFD). This kind of food would support the animals with 20% of calories from protein, the same calories from carbohydrates and 60% of calories from fat. The regimen of the diet continued for four weeks, after which the rats were ready for the study [44].

#### 2.12.4. Protocol of Animal Study

The study proceeded with five groups, each with five rats. The first group were kept as the non-obese control group (Group I) that kept on feeding with a standard commercial rodent diet. The other four groups were fed the HFD. The twenty obese rats that were fed the HFD were distributed among groups randomly. Group II served as the untreated obese rats. Group III obese rats received oral ATO suspension for 14 days (20 mg/kg). Group IV obese rats received oral NLC without ATO for 14 days. Finally, Group V obese rats received oral ATO-NLC for 14 days (20 mg/kg) daily. Animals received the investigated formulations by oral gavage. All obese rats under the investigation were continued on their regimen diet (HFD) throughout the whole period of the study. After two weeks of formulation administration, blood samples were collected and centrifuged for 10 min at 3000 rpm for serum separation. Serum samples were preserved at −20 °C for further analysis.

#### 2.12.5. Analysis of Biochemical Parameters

Blood serum was analyzed using specific kits, such as total cholesterol (T. Chol), low density lipoprotein (LDL), triglycerides (TG) and high-density lipoprotein (HDL) levels. These kits were used by following their protocols.

### 2.13. Statistical Analysis

Data were shown as the mean ± standard deviation (SD) for at least three experiments. Groups were considered to be significantly different from each other when *p* < 0.05. All statistical analyses were proven using SPSS statistics software, version 9 (IBM Corporation, Armonk, NY, USA). One-way analysis of variance (ANOVA) was performed using Design-Expert version 12.0 software (Stat-Ease, Minneapolis, MN, USA).

## 3. Results

### 3.1. Preliminary Study

According to the miscibility test, the most suitable ratio between solid and liquid lipids was 2:8 since it provided a homogeneous, miscible mixture with suitable consistency and without any phase separation.

### 3.2. Validating of BBD Data

A matrix of 15 experimental runs was constructed following BBD software to help in developing the NLC formulations. The selected independent variables and their corresponding dependent responses for each ATO-NLC formulation are exemplified in Table 2. Statistical analysis for the data obtained was performed using ANOVA, which is essential for fitting the model of the design. It was distinguished that, in all responses, the model F-value is termed to be significant as it was 390.56 and 510.41 for Y_1_ and Y_2_, respectively. Additionally, *p*-values were less than 0.05, which indicate that most of the model terms were significant. Another parameter is the lack-of-fit F-value, which was not significant for all responses, as shown in Table 3. Non-significant lack-of-fit is good, as it help the model to fit the data.

### 3.3. Analysis of Dependent Variables (Y_1_ and Y_2_)

#### 3.3.1. Influence of the Independent Variables on Particle Size

**Figure 2:**
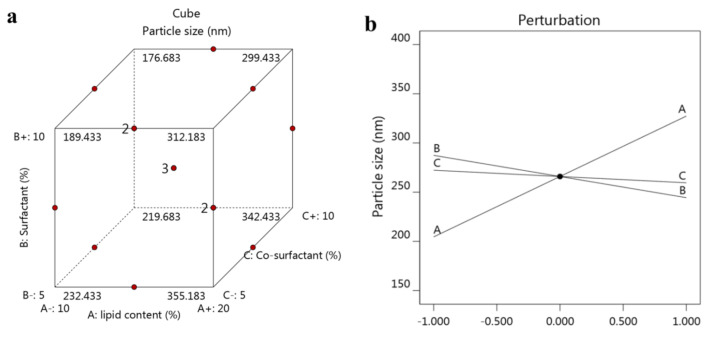
(**a**) Cube graph showing the predicted values and (**b**) perturbation plot displaying the influence of each independent variable alone on particle size Y_1_.

**Figure 3:**
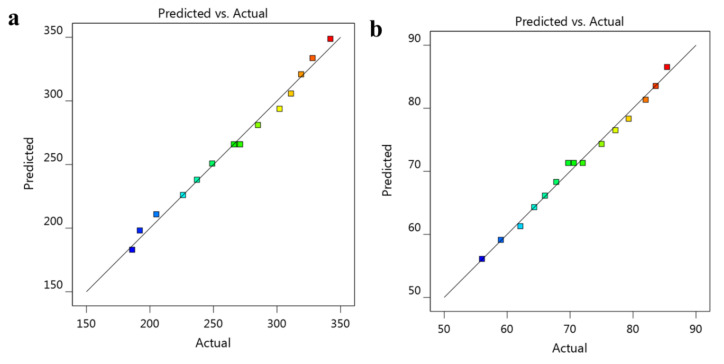
Linear correlation plot between predicted versus actual values for clarifying the influence of the lipid phase, surfactant and co-surfactant concentration on (**a**) particle size Y_1_ and (**b**) EE Y_2_.

As displayed in Table 2, the particle size of the developed ATO-NLC was evaluated and found to range between 186 ± 3.3 and 342 ± 4.5 nm with the polydispersity (PDI) value ranging between 0.13 ± 0.025 and 0.29 ± 0.042. The PDI value of all formulations indicates the homogeneity of the preparations and revealed that the distribution of size falls within a narrow range, which is considered a good sign for formulation stability [45]. Returning to Table 2, it is shown that the concentration of the lipid phase was directly proportional to the particle size of the formulation. Increasing lipid phase concentration would result in a similar increment in particle size. The rationale of this could be due to the expected coalescence of particles upon using higher lipid concentration that leads to larger size, in addition to a probable increase in the dispersed phase [46]. On the other side, upon using same lipid concentration, increasing the surfactant and co-surfactant could lower the particle size of the developed NLC. The reason behind this would be credited to the action of surfactant and co-surfactant in causing a substantial decrease in the interfacial tension between both phases that would contribute to forming droplets with smaller size [47]. Moreover, using the surfactant in higher concentration could participate in building a layer around the particles, behaving like a barrier that hinders the coalescence of the particles, keeping them small [48]. Illustrating the effect of the three independent variables A, B and C on the impact of particle size Y_1_ is clarified from Figure 2a showing a cube graph at which the predicted values are shown. Additionally, a perturbation plot was generated to explain the action of each independent variable on the dependent one. As obvious in Figure 2b, variable A showed a considerable influence on Y_1_ compared to other variables B and C. Moreover, the direction of the perturbation plot emphasized that the independent variable A exerted a synergistic effect on Y_1_, whereas other variables B and C had an antagonistic action. Furthermore, the linear correlation between observed particle size Y_1_ and the predicted one was highlighted as shown in Table 3 and Figure 3a, where the predicted R^2^ (0.9813) is in reasonable agreement with the adjusted one (0.9882). Yet, the following mathematical equation for Y_1_ was obtained and confirms the positive influence of independent variable A and the negative impact of B and C:

Y_1_ = 265.933 + 61.375 A − 21.5 B − 6.375 C

#### 3.3.2. Influence of the Independent Variables on EE

**Figure 4:**
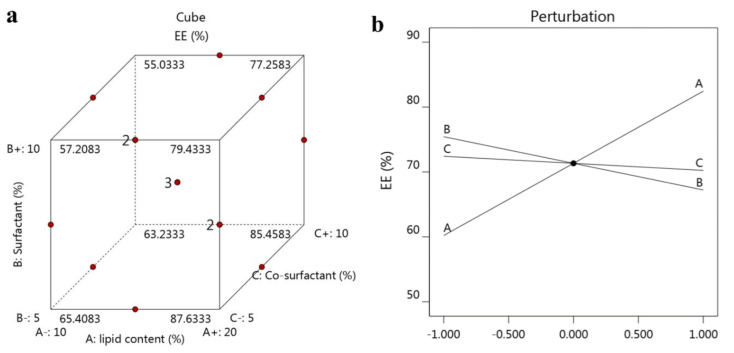
(**a**) Cube graph showing the predicted values and (**b**) perturbation plot displaying the influence of each independent variable alone on EE Y_2_.

The tendency of ATO to be entrapped into the NLC formulation was assessed via analyzing the % of EE. As per data presented in Table 2, the value of EE ranged from 56 ± 1.7 to 85.4 ± 2.4%. The interpretation of the data revealed that upon increasing lipid concentration, the amount of ATO entrapped into NLC would be increased as well. This is supposed to be due to the larger particle size obtained, which could accommodate for the larger amount of drug due to the availability of adequate space, which accordingly, enhances the drug EE [49]. The lipophilic nature of the drug represents a good point in enhancing its solubility in the melted lipid phase and, subsequently, easily entrapped [50]. Inversely, the increment of surfactant and co-surfactant would result in lowering the percentage of EE. This finding significantly correlates with particle size. Lower concentration of surfactant and co-surfactant provide smaller particles, which could not provide accommodations for a large amount of drug to be entrapped. Figure 4a shows a cube graph illustrating the predicted values of response, while Figure 4b depicts the perturbation plot signifying the effect of A, which provides an inverse relation with Y_2_; however, the influence of other variables B and C was less prominent, providing synergistic influence. Likewise, linear relation between predicted and actual values was detected as illustrated in Table 3 and Figure 3b, since the value of predicted and adjusted R^2^ were very close to each other (0.9879 and 0.9909), respectively. Similarly, the mathematical equation promoted our findings as follow:

Y_2_ = 71.3333 + 11.1125 A − 4.1 B − 1.0875 C

### 3.4. Data Optimization

**Figure 5:**
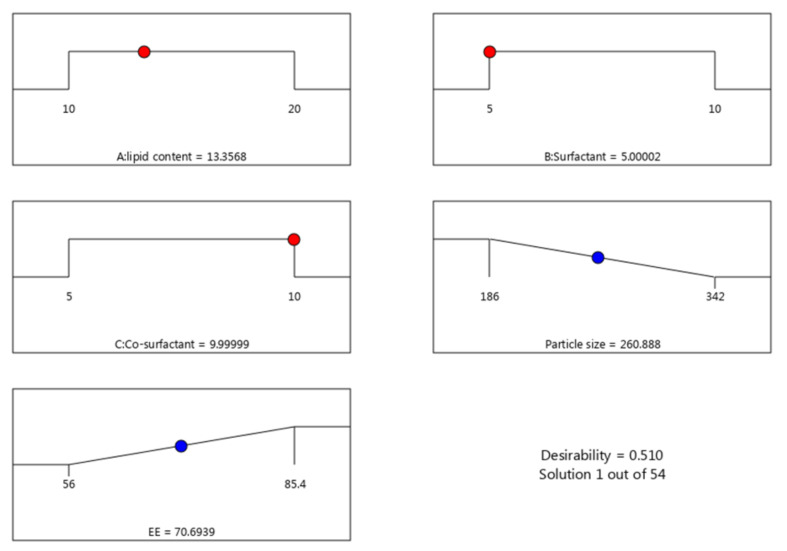
Optimization ramps for the investigated independent variables with their predicted dependent responses and the desirability value.

Numerical optimization and the desirability function support the selection of the optimized formula based on certain proper constraints. Guiding the responses toward certain desirable goals is the aim of the optimization process. The goals were adjusted to minimize the particle size and maximize the EE. Based on the prior adjustment, the concentration of the lipid phase was directed to be 13.35%, surfactant was 5% and co-surfactant 10%. Next, the design supposed some values for the independent variables that are anticipated to provide higher desirability value (0.510) as shown in Table 4 and Figure 5. Following such considerations, the optimized ATO-NLC formulation was prepared and compared with the observed calculated values. Conspicuously, a great close was identified between the predicted and the observed values.

### 3.5. Zeta Potential Measurment

**Figure 6:**
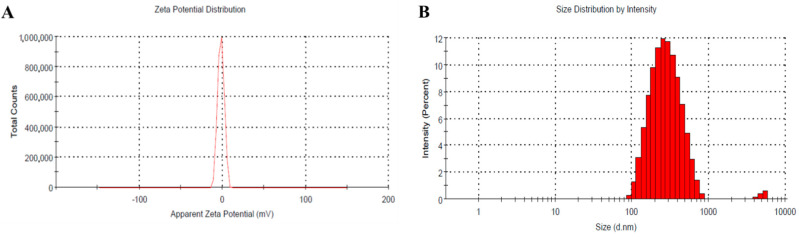
(**A**) Zeta potential and (**B**) particle size of optimized ATO-NLC formulation.

Zeta potential of the optimized ATO-NLC was determined in order to measure the surface electrical charge. As per data illustrated in Figure 6A, it was noticable that the zeta potential of the examined formulation tends to be approximately neutral, recording −1.77 mV. This could be illustrated on the basis of the surface modification of the formulation with PEG-DSPE that form a layer around the particles, acting as a stabilizing agent. This could be ascribed to increasing the hydrophilicity of the formulation by the inclusion of PEG-DSPE into NLC, reducing the overall charge (zeta potential) on the surface of the formulation. Therefore, it would prevent the aggregation and enhance the stability [51]. Much of the literature supports our findings since the surface modification of the particles shifted the charges to neutrality [52]. Curiously, this result certifies the physical stability of the formulation that would be further emphasized via the stability test. Additionally, Figure 6B, refers to the particle size of the optimized ATO-NLC formulation (254.2 ± 3.9) with its corresponding PDI value of 0.207 ± 0.19.

### 3.6. Fourier-Transform Infrared Spectroscopy (FTIR) Study

**Figure 7:**
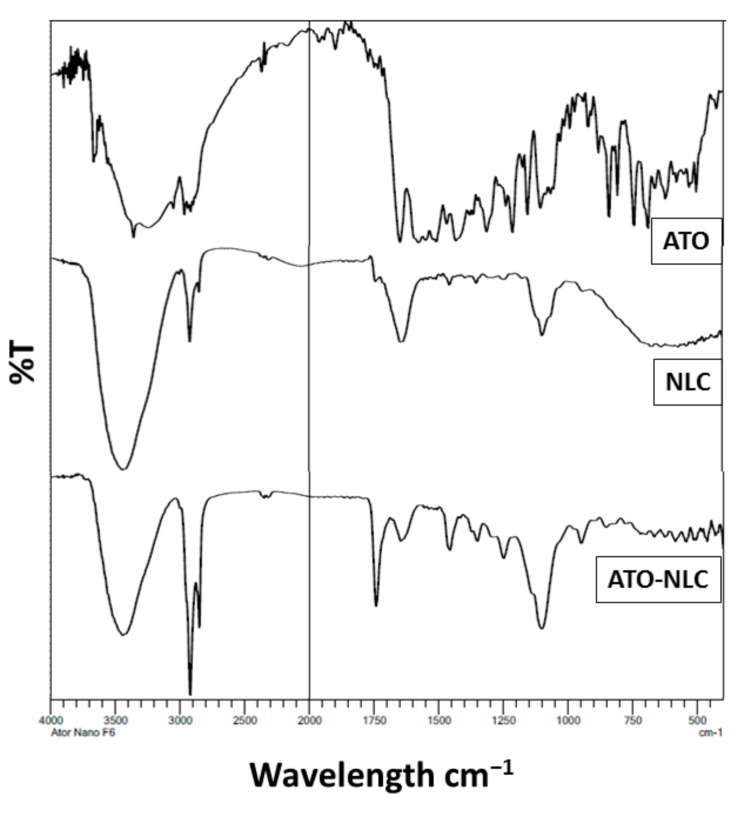
FTIR spectra of pure ATO, free NLC and optimized ATO-NLC formulation.

FTIR analysis was performed in order to identify any interaction that could happen between ATO and other excipients in the formulation as depicted in Figure 7. It was visible that the spectrum of pure ATO displayed a sharp peak related to the OH non hydrogen bond at 3660 cm^−1^, distinctive NH stretching at 3360 cm^−1^ and CH stretching at 2960 cm^−1^. Moreover, CO stretching was detected at 1650 cm^−1^, NH bending at 1570 cm^−1^, OH bending and CO stretching at 1430 and 1380 cm^−1^, respectively [53]. In contrast, upon examining the ATO-NLC spectrum, it was found that there was a decrease in the intensity of peaks at 3500 cm^−1^ and augmentation of aliphatic CH at 2900 cm^−1^, which concludes the type of non-polar attraction between the non-polar part of the drug and the lipid. Additionally, there was an increase in sharpness and decrease in the intensity of CO peaks that involved H bonding with OH groups. To conclude, it was observed that the characteristic peaks of NLC was present in ATO-NLC; additionally, peaks of ATO were present in ATO-NLC but the intensity of the peaks was changed, which indicates that ATO was dispersed into the lipid phase.

### 3.7. In Vitro Release

**Figure 8:**
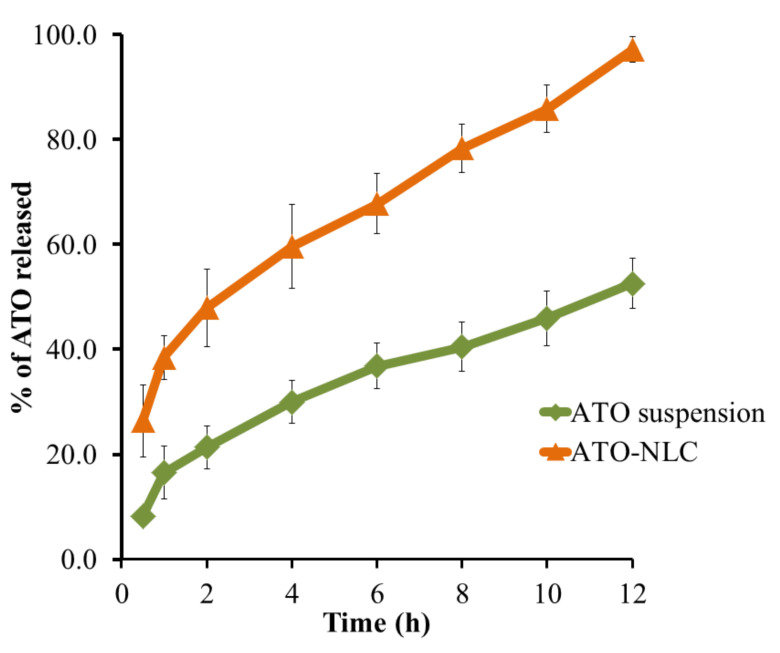
Outline of ATO in vitro release from optimized NLC formulations and suspension.

The release of ATO from the developed NLC was examined, and the outline of the in vitro study is apparent in Figure 8. The study was conducted by comparing the release of ATO from the optimized NLC and ATO suspension. It was obvious that there was a regular increase in the pattern of the release with time, since the % of ATO released reached its maximum after 12 h to be 97.17 ± 2.4%. As noted in the figure, the ATO release pattern followed a biphasic outline followed by a slower rate of release. The rapid release of ATO at the first 2 h could be attributable to a certain amount of ATO remaining at the outer shell of the formulation causing it to be released quickly and easily [32]. Nevertheless, the slower rate of release continued until 12 h to reach 97.17%, which is apparently owing to higher ATO lipophilicity, which preferred to be in the lipid core, so it would diffuse more slowly [54]. In contrast, ATO release from suspension was observed to follow a slower rate of release after 12 h as it reached 52.6 ± 4.8%. This could be returned to the availability of ATO in its crystalline nature, which is proven to be more stable and less soluble [55].

### 3.8. Kinetic Study

As can be observed from values given in Table 5, the coefficient (R^2^) related to different kinetic modeling was identified. The R^2^ value helps in determining the proper mechanism by which ATO was released from the formulations. In the current study, it was noted that ATO released from suspension followed the Korsmeyer–Peppas mechanism, since the value of its R^2^ (0.9774) was the greatest compared to that in other kinetic models [56]. On the contrary, it was revealed that ATO obeyed the Higuchi model when released from the examined ATO-NLC formulation. This was certified by plotting the % of drug release against time and determining the coefficient (R^2^) value, which was found to be 0.9938. The results provided the most linear model with the greatest (R^2^) when compared to other kinetic models. Formulations that obeyed Higuchi kinetic modeling signified diffusion by a controlled process and verified the erosion from the lipid matrix [43]. This was in agreement with Pinheiro et al., who confirmed that the release from mannose NLC follows the Higuchi diffusion model [56]. Additionally, Sharif et al. stated that the release of Propranolol Hydrochloride from NLC followed the Higuchi mechanism [57].

### 3.9. Stability Study

**Figure 9:**
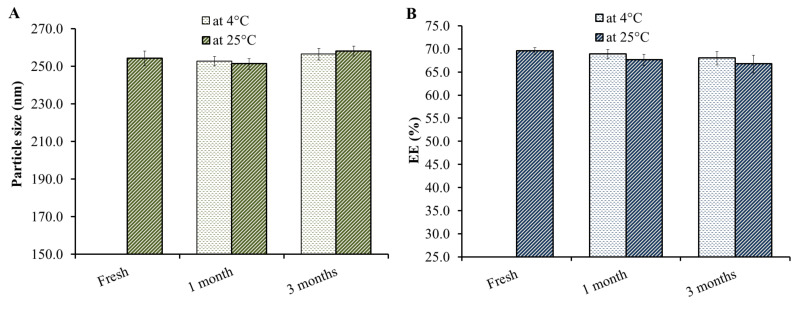
Stability study of ATO-NLC formulation after 25 ± 2 °C and 5 ± 2 °C for 1 and 3 months in terms of (**A**) particle size and (**B**) EE.

As perceived in the data of Figure 9, the stability of the ATO-NLC formulation with respect to particle size and EE was performed. The study was conducted over 3 months upon storing the formulation for 1 and 3 months under two different conditions; 25 ± 2 °C and 5 ± 2 °C. It could be established that ATO-NLC showed non-significant difference (*p* < 0.05) in terms of the evaluated parameters, particle size and EE following 1 and 3 months of storage under both conditions, 25 ± 2 °C and 5 ± 2 °C. The result confirmed the stability of the formulation that was previously investigated in zeta potential measurements. The great stability of the formulation definitely ascribed surface modification with PEG-DSPE, which plays a key role in avoiding the aggregation of particles, thus, keeping particle size and EE significantly the same, similar to a fresh preparation [53].

### 3.10. Evaluation of the Biochemical Parameters

Certain parameters provided an indication for the lipid profile status in the body and were evaluated and data are described in Table 6. As mentioned in the experimental protocol, one group was kept as a non-treated control, whereas the other four groups were kept on an HFD diet to render them obese. It was observed that groups of rats fed on HFD for a period of 4 weeks recorded a significant increment in the levels of their T-Chol, LDL and TG (*p* < 0.05), indicating the induction of hyperlipidemia [32]. Additionally, a significant decline was detected in their HDL levels (*p* < 0.05) when compared to the non-obese control group (Group I). Furthermore, Group III, IV and V treated with ATO suspension, free NLC and ATO-NLC, respectively, exhibited a significant reduction in the level of T-Chol, LDL and TG when compared to the non-treated obese group (Group II) (*p* < 0.05). This is in accordance with Ghanem et al., who established that ATO suspension and the developed ATO-NLC resulted in a reduction in the same evaluated parameters when compared to the non-treated obese group [31]. As is clear from the data, Group IV exhibited a significant reduction in the level of T-Chol, LDL and TG, which could emphasize the effect of oil as a hypolipidemic agent [18,58]. Moreover, the ATO-NLC treated group (Group V) demonstrated a significant reduction in T-Chol, LDL and TG levels when compared to Group III and Group IV (*p* < 0.5). To sum up, the obtained data could highlight the effective role of olive oil used in the development of an NLC formulation. Further, olive oil could enhance the pharmacological behavior of ATO when entrapped into an NLC formulation. Prominently, HDL provided a non-significant change in Group III and IV comparable to the non-treated group (Group II) (*p* < 0.5), which could be accredited to the short period of treatment that lasted for only 14 days. Observably, the HDL value exhibited the highest elevation in Group V treated with the ATO-NLC formulation, revealing its dominant effect. Our finding was in agreement of Elmowafy et al., since the formulated ATO-NLC provides the most remarkable increase in levels of HDL in animal models [32]. Of note, treatment with ATO-NLC could restore all the evaluated biochemical parameters to normal values similar to observations of Group I (non-obese control group), indicating the efficiency of the developed formulation.

## 4. Conclusions

The objective of the current investigation was to develop a nanolipid formula that could enhance the drug solubility and efficiently achieve its pharmacological activity. To that end, nanostructured lipid carrier formulations incorporating a hypolipidemic drug (ATO) prepared with olive oil were developed. The optimized formula was nominated by operating one of the QbD platforms. The optimized ATO-NLC exhibited proper characteristics, including particle size, zeta potential and EE. It revealed an extended in vitro release following Higuchi kinetic modeling. Studying the stability of the formulation in varied conditions proved its high stability over a period of three months. Eventually, the ATO-NLC formulation displayed a significant reduction in the evaluated biochemical parameters establishing the significance of combining olive oil and ATO in an NLC formulation for enhancing in vivo anti-hyperlipidemic activity.

## Figures and Tables

**Table 1:** Data from BBD displaying the independent variables with their level of variation and the studied dependent variables.

Independent Variable	Symbol	Level of Variation		
		Lowest (−1)	Central (0)	Highest (1)
Lipid phase concentration (%)	A	10	15	20
Surfactant concentration (%)	B	5	7.5	10
Co-surfactant concentration (%)	C	5	7.5	10
**Dependent variable**	**Symbol**	**Constraints**		
Particle size (nm)	Y_1_	Minimize		
EE (%)	Y_2_	Maximize		

**Table 2:** Experimental runs for different ATO-NLCs created by BBD and their observed dependent variables.

Formula	Independent Variables			Dependent Variables		PDI
	A (%)	B (%)	C (%)	Y_1_ (nm)	Y_2_ (%)	
**F1**	20	10	7.5	311 ± 4.6	79.3 ± 1.7	0.29 ± 0.027
**F2**	15	7.5	7.5	270 ± 3.6	69.7 ± 2.0	0.23 ± 0.030
**F3**	20	5	7.5	342 ± 4.5	85.4 ± 2.4	0.28 ± 0.025
**F4**	15	5	10	285 ± 4.1	75.0 ± 2.9	0.24 ± 0.050
**F5**	20	7.5	5	328 ± 4.9	83.6 ± 2.6	0.22 ± 0.045
**F6**	10	7.5	10	192 ± 3.3	59.0 ± 1.8	0.13 ± 0.025
**F7**	10	7.5	5	205 ± 2.6	62.1 ± 2.8	0.17 ± 0.031
**F8**	15	10	5	249 ± 4.2	67.8 ± 2.4	0.20 ± 0.031
**F9**	15	5	5	302 ± 2.5	77.2 ± 2.3	0.26 ± 0.035
**F10**	10	5	7.5	226 ± 3.6	64.3 ± 2.8	0.24 ± 0.053
**F11**	10	10	7.5	186 ± 3.3	56.0 ± 1.7	0.19 ± 0.040
**F12**	15	7.5	7.5	271 ± 3.8	70.6 ± 2.4	0.23 ± 0.031
**F13**	15	7.5	7.5	266 ± 4.3	72.0 ± 2.1	0.23 ± 0.049
**F14**	20	7.5	10	319 ± 2.8	82.0 ± 1.9	0.29 ± 0.042
**F15**	15	10	10	237 ± 3.6	66.0 ± 2.3	0.22 ± 0.035

A: Lipid phase concentration; B: Surfactant concentration; C: Co-surfactant concentration; Y_1_: Particle size; Y_2_: EE.

**Table 3:** Statistical analysis of model and fit statistic for all responses.

Source	Y_1_		Y_2_	
	F-Value	*p*-Value	F-Value	*p*-Value
**Model**	390.56	<0.0001 *	510.41	<0.0001*
A	1033.69	<0.0001 *	1336.51	<0.0001 *
B	126.85	<0.0001 *	181.93	<0.0001 *
C	11.15	0.0066 *	12.80	0.0043 *
Lack-of-Fit	4.87	0.1820	0.4503	0.8355
R^2^ analysis				
R^2^	0.9907		0.9929	
Adjusted R^2^	0.9882		0.9909	
Predicted R^2^	0.9813		0.9879	
Adequate Precision	59.4466		68.5291	

A: Lipid phase concentration; B: Surfactant concentration; C: Co-surfactant concentration; Y_1_: Particle size; Y_2_: EE; and *, significant.

**Table 4:** Predicted and observed value for the optimized ATO-NLC formulation.

Selected Independent Variables	Constraint	
Lipid phase concentration (%)	In range	
Surfactant concentration (%)	In range	
Co-surfactant concentration (%)	In range	
Response	Predicted values	Observed values
Particle size (nm)	260.88 ± 5.39	254.23 ± 3.9
EE (%)	70.69 ± 0.49	69.56 ± 0.70

**Table 5:** Different kinetic modeling for illustrating ATO release from suspension and NLC.

Kinetic Model	ATO Suspension	ATO-NLC
Zero order kinetic (R^2^)	0.9574	0.9669
First order kinetic (R^2^)	0.8018	0.8736
Higuchi kinetic (R^2^)	0.9763	0.9938
Korsmeyer–Peppas kinetic (R^2^)	0.9774	0.9909

**Table 6:** The evaluated biochemical parameters following treatment with ATO formulations.

Animal Groups	TG (mg/dL)	T-Chol (mg/dL)	HDL (mg/dL)	LDL (mg/dL)
Group I-Non obese	76.18 ± 2.49	114.86 ± 3.73	41.26 ± 1.89	60.32 ± 2.74
Group II-Obese	124.54 ± 5.16 ^#^	141.36 ± 4.76 ^#^	36.94 ± 0.94 ^#^	81.34 ± 5.50 ^#^
Group III (ATO Suspension)	86.02 ± 3.70 *	123.04 ± 3.08 *	38.10 ± 0.91	63.34 ± 1.38 *
Group IV (Free NLC)	106.42 ± 3.89 *	127.90 ± 2.75 *	37.04 ± 0.68	74.30 ± 2.42 *
Group V (ATO-NLC)	76.78 ± 3.38 * ^$^	118.98 ± 1.38 * ^$^	39.40 ± 1.39 *	59.72 ± 1.53 * ^$^

Results are expressed as mean value ± SD, where *n* = 5. ^#^ *p* < 0.05, compared to Group I, * *p* < 0.05, compared to Group II, and ^$^ *p* < 0.05, compared to Group III and IV.

## Data Availability

Not applicable.
